# Organocatalyzed Synthesis of [3.2.1] Bicyclooctanes

**DOI:** 10.3390/molecules23051039

**Published:** 2018-04-28

**Authors:** Ignacio E. Tobal, Alejandro M. Roncero, Narciso M. Garrido, Isidro S. Marcos, David Díez

**Affiliations:** Departamento de Química Orgánica, Universidad de Salamanca, Plaza de los Caídos 1–5, 37008 Salamanca, Spain; ignaciotobal@usal.es (I.E.T.); alexmaron@usal.es (A.M.R.); nmg@usal.es (N.M.G.); ismarcos@usal.es (I.S.M.)

**Keywords:** organocatalysis, bicyclo[3.2.1], bicycle

## Abstract

Organocatalysis constitutes one of the main research areas in organic chemistry from the last two decades. This chemistry has been applied to the synthesis of many natural products and structures in a manner that reduces the residues and so the ecological impact. In this review, we consider the work that has been done for the synthesis of bicyclo[3.2.1]octane framework. This structure is present in many natural products with very important biological activities.

## 1. Introduction

**Figure 1:**
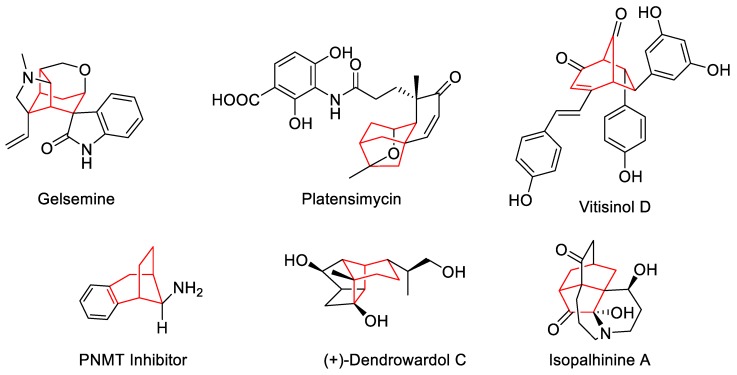
Biologically active [3.2.1] bicycle compounds.

There is a growing interest in organic chemistry on the synthesis of compounds with a [3.2.1] framework, due to the presence of this moiety in molecules with biological interest, for example the natural products gelsemine, platensimycin, and vitisinol D, or synthetic products as the PNMT (phenylethanolamine *N*-methyltransferase) inhibitor (Figure 1). Gelsemine has attracted considerable attention since its discovery due to its specific antinociception in chronic pain [1,2]. In the same manner, platensimycin possesses a potent and broad-spectrum Gram-positive antibacterial activity, with no cross-resistance to an array of major antibiotic-resistant microbes and has been synthesized several times [3,4]. Vitisinol D, which has not been synthesized in enantiomeric form and only one racemic total synthesis has been described [5], presents antithrombotic properties [6], and finally compounds that are inhibitors of phenylethanolamine *N*-methyltransferase as PNMT inhibitor [7] are shown in Figure 1. Recently, the [3.2.1] bicyclic moiety has been included in more complex synthetic structures as (+)-dendrowardol C [8] and isopalhinine [9], a fact that has awaken the interest of researchers. Due to this special attention in recent years, important contributions to the synthesis of this framework have been reported, not only for the total synthesis of natural compounds that contain it, but also for the synthesis of the structure itself.

There are several reviews on this subject, as the excellent reviews of Rodriguez et al. [10,11]. Taking in to account these reviews, the reactions appeared in the literature for the synthesis of the bicyclo[3.2.1] systems using organocatalysis from January 2013 to February 2018 and they are considered in this work.

**Figure 2:**
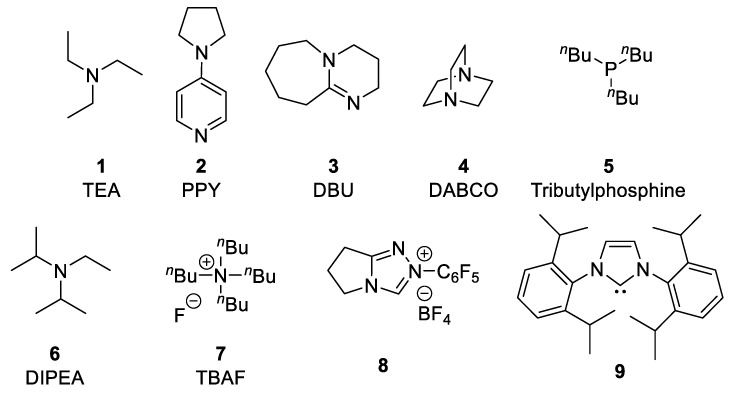
Non-chiral organocatalysts **1**–**9**.

In this review, we centred the attention to the synthesis of these compounds by organocatalytic methods. Organocatalysis is perhaps the most developed organic chemistry area in the last 20 years [12]. This review considers the synthesis by organocatalysis of the bicyclo[3.2.1]octane scaffold using non-chiral and chiral organocatalysts; although the main interest of using organocatalysis is the synthesis of chiral compounds. However in recent years, the interest on the use of organocatalysis in non-asymmetric synthesis is rising [13]. The non-chiral catalysts (**1**–**9**) that appear throughout this review are shown in Figure 2. A non-chiral triazolium salt (**8**) has been added in this figure as it was used for the synthesis of the bicyclic system. The chiral triazole salts have been previously used in order to achieve high enantioselectivity in an intramolecular Stetter reaction by Rovis et al. [14].

**Figure 3:**
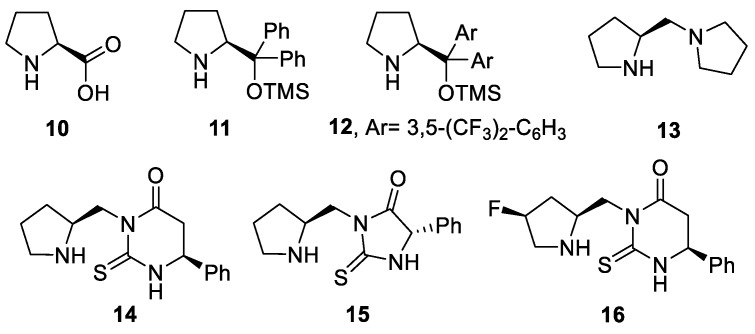
Chiral organocatalysts: proline **10** and derivatives **11**–**13**, imidazolidone **15** and pyrimidones **14** and **16**.

The importance of organocatalysis started with the seminal papers of List et al. on the use of proline as catalyst in order to obtain non-racemic aldol compounds [15], and the one of MacMillan et al. on the use of chiral imidazolidinones for the enantioselective Diels-Alder reaction [16]. Proline **10,** proline derivatives **11**–**13**, second generation MacMillan’s catalyst imidazolidone **15**, and pyrimidinones as **14** and **16** employed in chiral syntheses of the bicyclic moiety are included in Figure 3.

**Figure 4:**
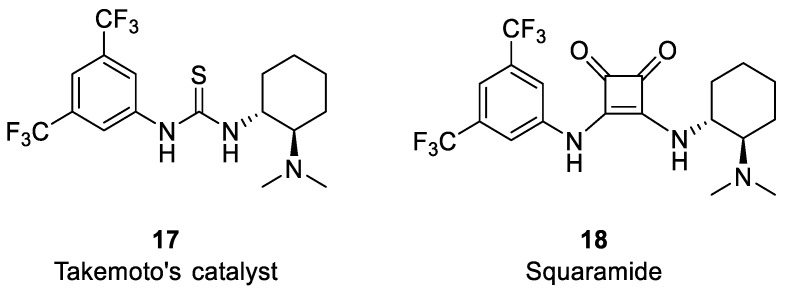
Cyclohexanediamine organocatalysts: Takemoto’s organocatalyst **17** and squaramide **18**.

Other organocatalysts, such as Takemoto’s catalyst **17,** with a cyclohexanediamine-derived amino thiourea scaffold, provide high enantioselectivity for the Michael addition [17] and has been widely used in organocatalysis (Figure 4).

**Figure 5:**
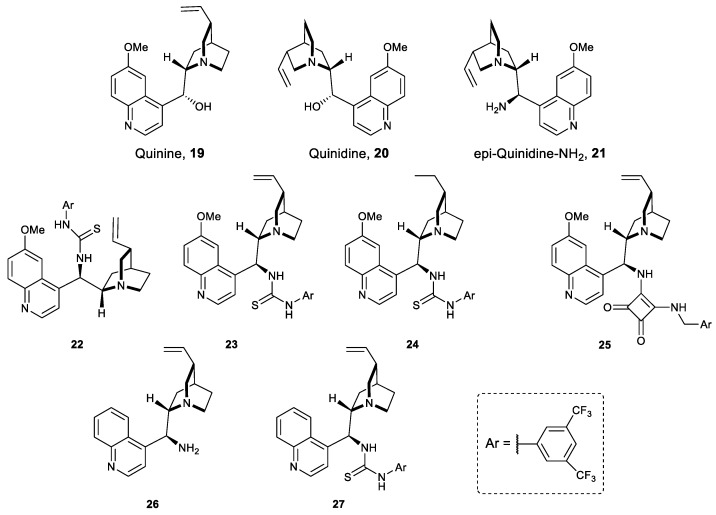
Quinine **19**, quinidine **20,** and derived organocatalysts **21**–**27**.

The asymmetric dihydroxylation of olefins by chiral complexes of dihydroquinine and dihydroquinidine derivatives by Hentges and Sharpless [18] opened the door to the use of quinine and quinidine derivatives not only in asymmetric synthesis, but also as chiral bases in organocatalysis [19]. The structures of quinine, quinidine, and other derivatives are shown in Figure 5.

## 2. Non-Chiral Acids or Bases for the Synthesis of Bicyclo[3.2.1]Octanes

**Scheme 1:**
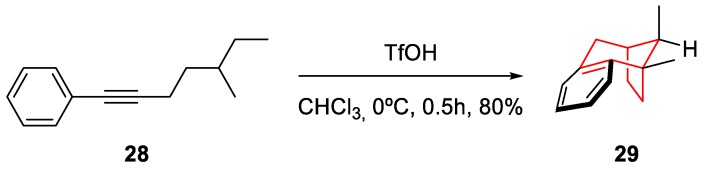
Acid-promoted rearrangement of **28** for [3.2.1] bicyclic system formation, **29**.

Organic acid catalysts are more restricted than bases, and are mainly related with rearrangements [20], as can be seen in the acid-induced protonation by TfOH and the subsequent rearrangement to form the bicyclo[3.2.1] system, **29** (Scheme 1) [21]. Su et al. tested the conditions and realized the scope of this reaction.

**Scheme 2:**
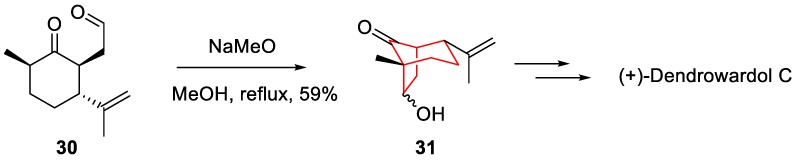
Base-promoted cyclization of 1,4-diketone **30** yielding the bicyclo[3.2.1] intermediate **31**.

Among the non-chiral bases used for the synthesis of bicyclo[3.2.1]octane scaffold are not only the non-chiral bases of Figure 2, but other as sodium methoxide or inorganic bases of this kind have been widely used in the literature, see for example Carreira et al. (Scheme 2) [8].

An excellent approach to bicyclo[3.2.1]octane in *ent*-kaurenoids has been published recently [22,23], where the use of acids or bases for the synthesis of these systems can be seen. In an *N*-heterocyclic carbene catalyzed cleavage of vicinal diketones, the bicyclo[3.2.1] system was obtained as a side product, mainly when diisopropylamine was used [23].

### 2.1. Synthesis of Platensimycin Core

**Scheme 3:**
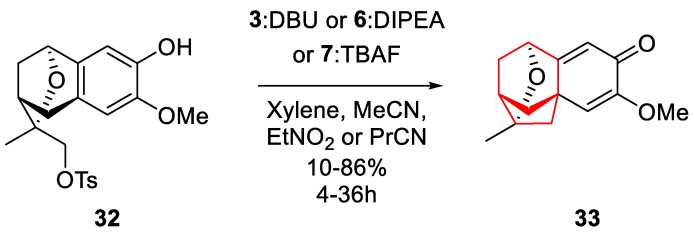
Organocatalyzed cyclization reaction from **32** to **33** to achieve platensimycin core.

Platensimycin is a potent fatty acid synthase inhibitor. Many efforts have been devoted to the synthesis of its cage-like ketolide core. The work of Eey and Lear [4], which can be considered a base-promoted alkylation, uses DBU **3**, DIPEA **6**, and TBAF **7** as bases (Scheme 3). The best result was obtained with **6** and xylene as solvent.

### 2.2. Synthesis with Chromone Derivatives

**Scheme 4:**
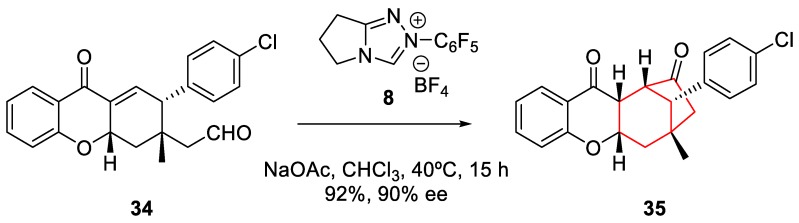
Stetter reaction of **34** for the synthesis of the bicyclic system: **35**.

Chen et al. made a chiral tricyclic compound by organocatalysis, but the cyclization of **34** to obtain the bicyclo[3.2.1] core was made using the triazolium salt **8** that produces an intramolecular Stetter reaction, obtaining **35** with exclusive diastereocontrol (Scheme 4) [24].

### 2.3. Synthesis of the Bicyclo[3.2.1] from 1,4-Cyclohexanedione

**Scheme 5:**
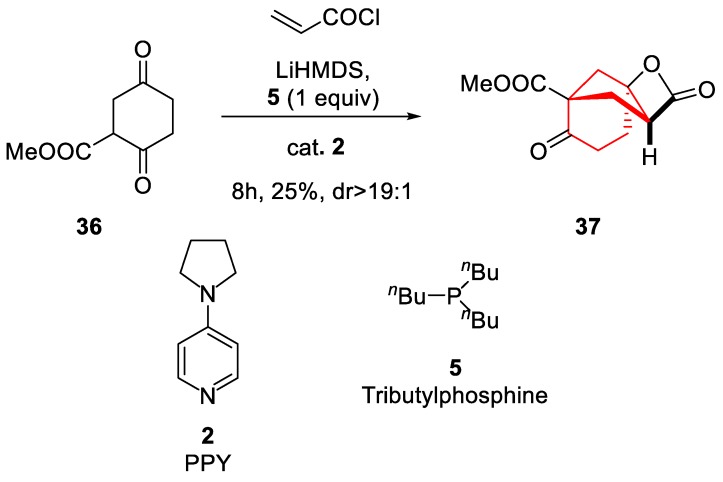
Synthesis of the bicyclo[3.2.1] system (**37**) from **36** using PPY **2** as catalyst.

Romo et al. were able to synthesize the required bicyclic fragment, as shown in Scheme 5 [25]. This synthetic methodology is a variant of the previously reported procedure by Zhong et al. [26]. It is a multicomponent reaction that uses α,β-unsaturated acylammonium intermediates generated by activation of unsaturated acyl chlorides that cycle to form the bicyclo[3.2.1]octane fragment, **37**.

### 2.4. Use of Oxo Michael Reaction for the Synthesis of the Bicyclo[3.2.1]

**Scheme 6:**
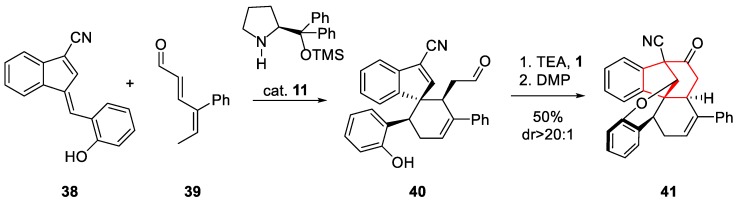
Oxo-Michael-aldol cascade reaction of **38** and **39** using **11** as organocatalyst achieving **40**, that can cycle in basic media yielding the bicyclic system: **41**.

Jørgensen et al. in their organocatalytic synthesis of chiral spiroindenes by trienamine catalysis, carried out treating an achiral phenol with triethylamine **1** obtained the bicyclic system **41** [27]. As can be seen in Scheme 6, the mechanism can be understood as an oxo-Michael-aldol cascade reaction.

### 2.5. Use of Intramolecular Michael Reaction for the Synthesis of the Bicyclo[3.2.1]

**Scheme 7:**
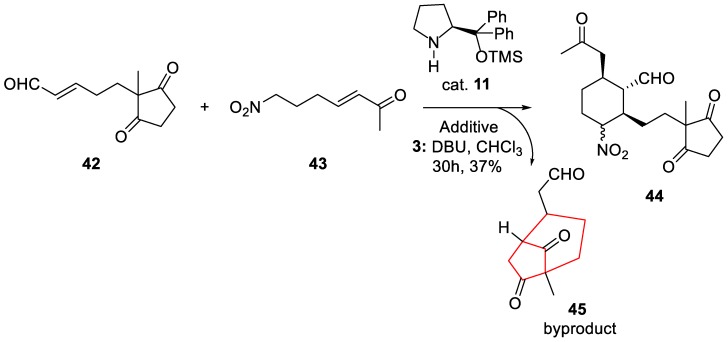
Organocatalyzed reaction of **42** and **43** by **11** produces **44**, but the reaction intermediate can evolve via intramolecular Michael reaction yielding the byproduct **45** with the bicyclo[3.2.1] scaffold.

Lee et al., in their one pot organocatalytic enantioselective Michael-Michael-aldol-Henry cascade reaction, describe a byproduct (**45**) with the bicyclo[3.2.1] scaffold in some conditions, as shown in Scheme 7 [28]. When the Hayashi-Jørgensen catalyst (**11**) is used in the presence of some bases as DBU (**3**) or other additives in chloroform, the bicyclic compound **45** is obtained in a non-chiral form as major product. This reaction can be understood as an intramolecular Michael reaction.

**Scheme 8:**
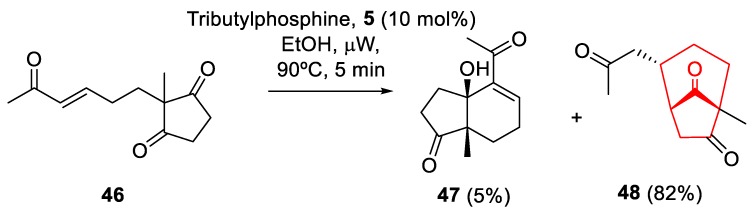
The intramolecular Michael reaction of **46** under microwave activation conduces to the formation of the bicycle **48** and the side-product **47**, where a single cyclization has occurred.

Another intramolecular reaction has been carried out by Miesch et al. [29]. In this case, the intramolecular reaction is done with an adequate functionalized cyclopentanone and using tributylphosphine **5** as base under microwave activation, obtaining **48** in good yield, Scheme 8.

**Scheme 9:**
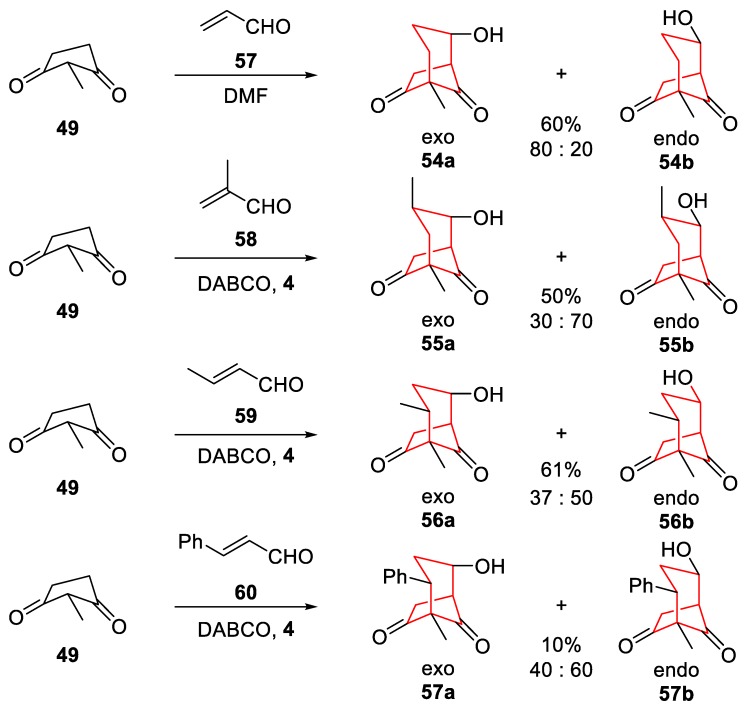
Synthesis of bicycloketols (**54**–**57**) synthesized from methylcyclopentanodione (**49**) and acrolein (**57**) or derivatives (**58**–**60**).

Marson et al. have recently described studies in the domino Michael-aldol annulation for the stereocontrolled synthesis of bicyclo-ketol derivatives (Scheme 9) [30]. The authors observed that in the case of bicyclo[3.3.1]nonane diones the *exo*-ketol is generally preferred over the *endo*-ketol. In the case of bicyclo[3.2.1]octane diones, an α-methyl group change in the Michael acceptor modifies the preference to the *endo*-alcohol (**54**–**55**). However, β-methyl or phenyl substituent exerts a weaker effect (**56** and **57,** respectively). The reaction between the diketone **49** and the aldehyde **52** catalyzed by **4** yields the exo- (**56a**), endo-hydroxybicyclo[3.2.1]octane (**56b**) and a side product in a 37:50:15 ratio, with a global yield of 61% for the pair of bicyclic products **56a** and **56b**.

### 2.6. Synthesis of the Bicyclo[3.2.1] by Isomerization of Spirocyclic Compounds

**Scheme 10:**
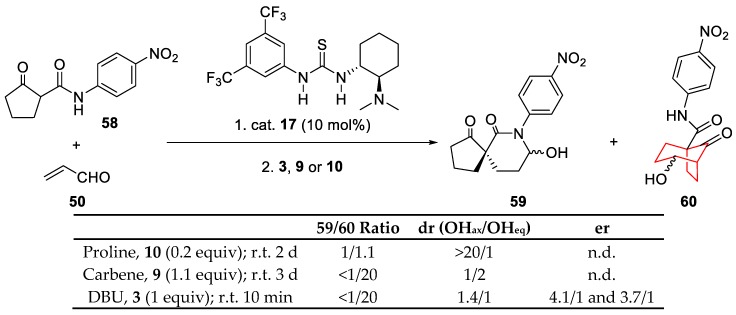
The organocatalyzed reaction of **50** and **58** conduces to a product mixture that shows an equilibrium between a spirocycle (**59**) and the bicyclo[3.2.1]octane (**60**).

Rodriguez et al. treated 1,3-ketoamides with acroleine in the presence of Takemoto’s catalyst **17** obtaining a spirocyclic compound **59** that was in equilibrium with the [3.2.1] bicyclic compound **60** as shown in Scheme 10 [31].

**Scheme 11:**
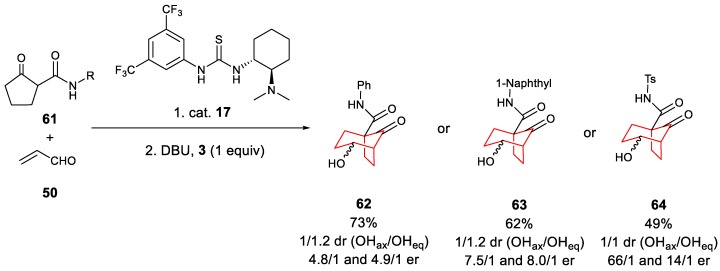
Organocatalyzed reaction of acrolein (**50**) and 1,3-ketoamide (**61**) by Takemoto’s catalyst, **17** to afford [3.2.1] bicyclic system.

The authors indicated that there is an equilibrium between the kinetic compound **59** and the thermodynamic one **60**. The authors established that Takemoto’s catalyst **17** is involved in the process as no interconversion between the products was obtained in its absence. The addition of a catalytic amount of proline **10** had limited impact. Stoichiometric addition of carbene **9** or DBU **3** shifted the equilibrium towards the bicyclo[3.2.1] compound. Moderated to good results were achieved in the obtention of the bicyclic compounds **62**–**64** when DBU **3** was used (Scheme 11).

### 2.7. Intramolecular Diels-Alder Reaction

**Scheme 12:**
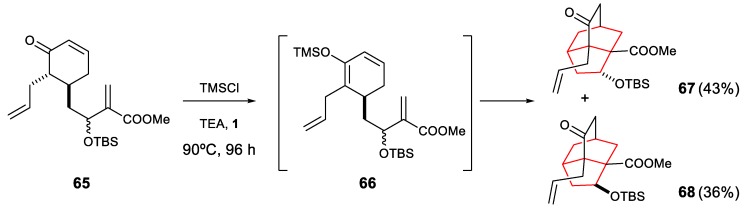
Diels-Alder reaction applied to the preparation of bicyclo[3.2.1]octane. The α,β-unsaturated ketone **65** reacts with TMSCl achieving the trimethylsilylether derivative, **66**, that evolves via intramolecular Diels-Alder reaction forming the bicycle[3.2.1]octane: **67** and **68**.

Although this is not a strictly organocatalytic reaction, Rychbowsky et al. [9] described the synthesis of the bicyclic system (**67** and **68**) by the Diels-Alder reaction through a silylenolether, as shown in Scheme 12.

## 3. Chiral Organocatalysis for the Synthesis of Bicyclo[3.2.1]Octanes

Herein we report the chiral organocatalytic synthesis of bicyclo[3.2.1]octane. We begin this point with the enantioselective approaches to the bicyclic scaffold from cyclic precursors, according to the classification followed in the previous review by Rodriguez et al.

### 3.1. Enatioselective Approaches to Bicyclo[3.2.1]octanes from Five-Membered Rings

**Scheme 13:**
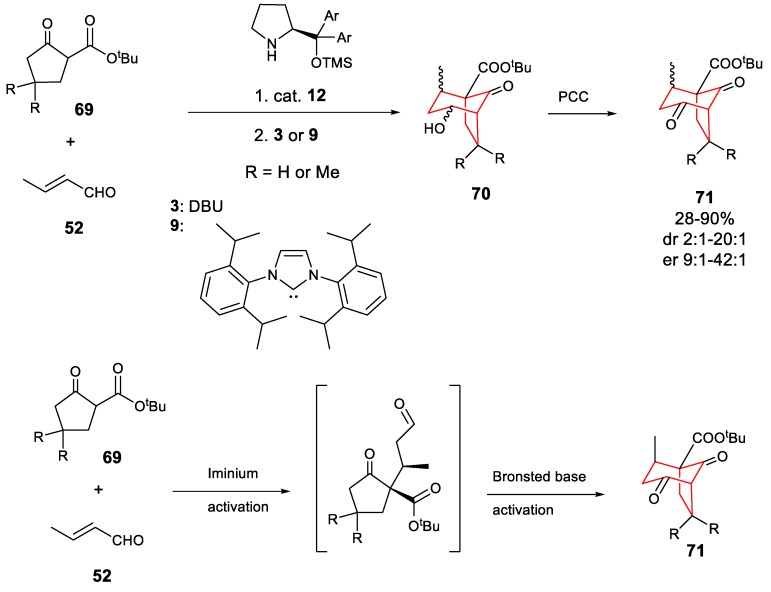
Amino and carbene catalysis for the synthesis of the bicyclic system from ketoesters (**69**) and crotonaldehyde **52**.

Recently, there has been an increasing interest in the use of dual catalysis. To this respect, the use of aminocatalysis and carbene catalysis is one of the most interesting ones [32]. Rodriguez et al., in their synthesis of suberosanone, studied the cyclization for the obtention of the [3.2.1] bicyclic system, see Scheme 13 [33].

The authors observed that imidazolydene *N*-heterocyclic carbenes like **9** are potent Brønsted bases able to catalyse Michael aldolization sequences with little tendency to reversibility. In this manner, the authors planned the synthesis of the bicyclo[3.2.1] by a sequential combination of the aminocatalysts **3** and **12** and the carbene **9**. They examined the reaction of a β-ketoester with crotonaldehyde **52** under various conditions. As the four possible diastereomers were obtained, the crude products were oxidized towards the corresponding diketones. The combination of catalyst **12** with DBU **3** gives the required products with good yield but low enatioselectivity. Conversely, the sequential addition of catalyst **12** and carbene **9** and subsequent oxidation produced good yields and stereoselectivities.

**Scheme 14:**
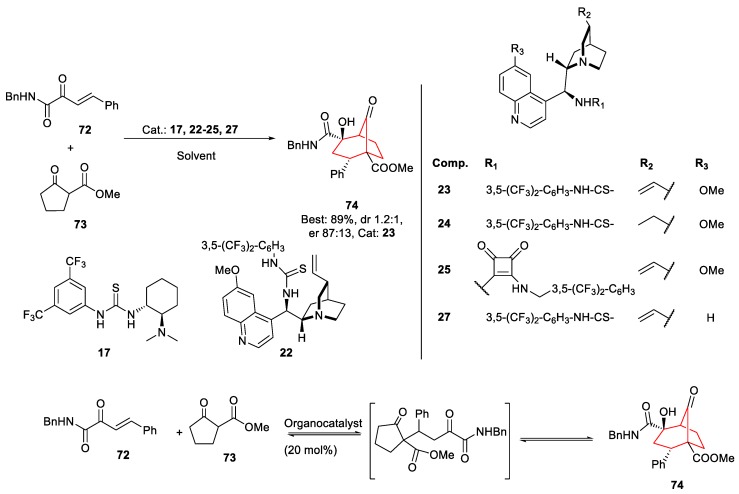
Organocatalytic domino Michael-aldol with β,γ-unsaturated amides (**72**) and ketoesters (**73**) yielding the bicyclo[3.2.1]octane scaffold: **74**.

Recently, the group of Alexakis described an organocatalytic domino Michael-aldol reaction of cyclic 1,3-ketoesters with β,γ-unsaturated amides affording chiral bicyclo[3.2.1]octanes [34]. They started the study with the reaction of (*E*)-*N*-benzyl-2-oxo-4-phenylbut-3-enamide with methyl cyclopentanonecarboxylate in toluene, catalyzed by Takemoto’s catalyst **17**. The desired bicyclic compound was obtained with 60% yield, excellent diastereoselectivity and enantioselectivity of 37%. When the organocatalyst was changed to a different quinine derived catalysts as **22**–**25** and **27**, good diastereselectivies and moderate enantioselectivities were obtained in all cases. The best catalyst was **24**, giving a yield of 75% with diastereoselectivity >20/1 and enatioselectivity of 54%. When the reaction was carried out in dichloromethane instead of toluene, the enantiomeric excess increased to 73% (Scheme 14).

**Scheme 15:**
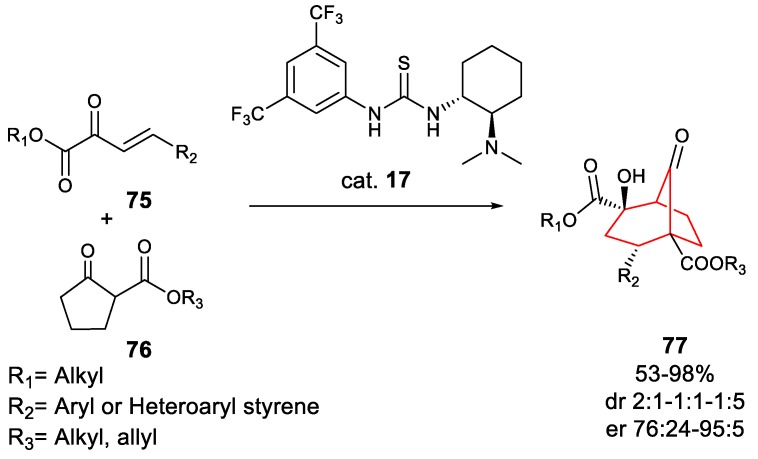
Organocatalytic obtention of the bicyclic system (**77**) via domino Michael-aldol reaction with β,γ-unsaturated esters (**75**) and 1,3-ketoesters (**76**).

The same group studied the reaction of cyclic 1,3-ketoesters with β,γ-unsaturated-1,2-ketoesters in presence of catalyst **17**, **22**, **23**, and **24**. They found that catalyst **17** was the one of election that can achieve the synthesis of polysubstituted chiral bicyclo[3.2.1]octanes in good diastereoselectivities and enantioselectivities and excellent yields (Scheme 15) [35].

**Scheme 16:**
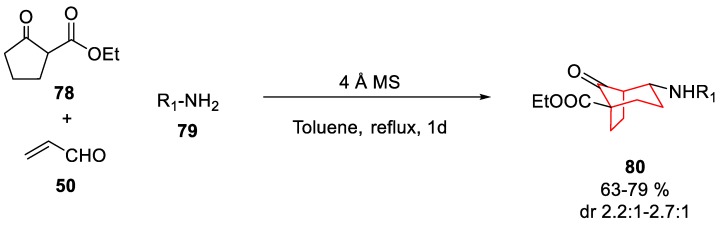
Three component reaction (α,β-unsaturated aldehyde, **50**; primary amine, **79**; and 1,3-ketoester, **78**) for the synthesis of bicyclic system: **80**.

Later on, Rodriguez described a reaction of ethyl cyclopentanonecarboxylate with acroleine in presence of different amines obtaining bicyclic systems with the amino function resulting in good yields and diastereoselections (Scheme 16) [36].

**Scheme 17:**
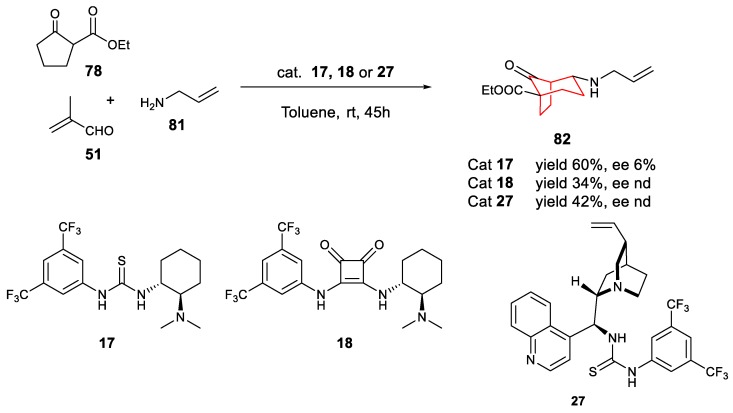
Three component reaction (α,β-unsaturated aldehyde, **51**; allyl amine, **81**; and 1,3-ketoester, **78**) for the synthesis of bicyclic system **82** with chiral organocatalysts.

Three component reactions have been described for the synthesis of these bicyclic systems (Scheme 17). The reaction conditions were studied to obtain enantioenriched compounds. When using organocatalyst **17**, **18**, and **27** the reaction produces only one diastereomer, although in very low enantiomeric excess.

**Scheme 18:**
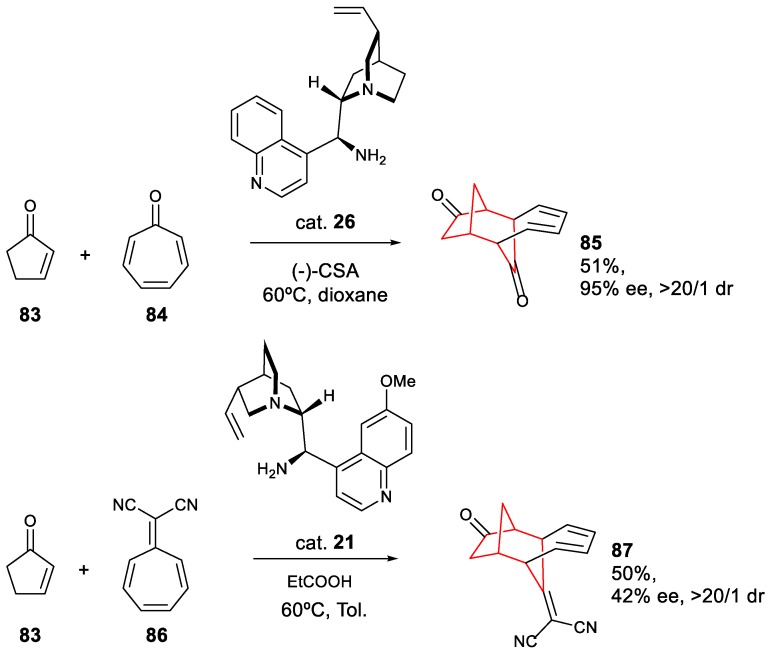
First organocatalyzed [6 + 4] cycloaddition reaction using the cyclic unsaturated ketones **83** and **84** or **86**, yielding the bicyclic products **85** and **87** respectively.

Very recently Jørgensen has described the first catalytic stereoselective intermolecular [6 + 4] cycloaddition (Scheme 18) [37]. This reaction is included into the so-called higher-order cycloadditions, excellent tools for solving synthetic challenges [37,38]. The novel organocatalytic asymmetric higher-order cycloaddition paves the way for further development in this area for the [3.2.1] bicyclic systems.

### 3.2. Enantioselective Approaches to Bicyclo[3.2.1]Octanes from Six-Membered Rings

**Scheme 19:**
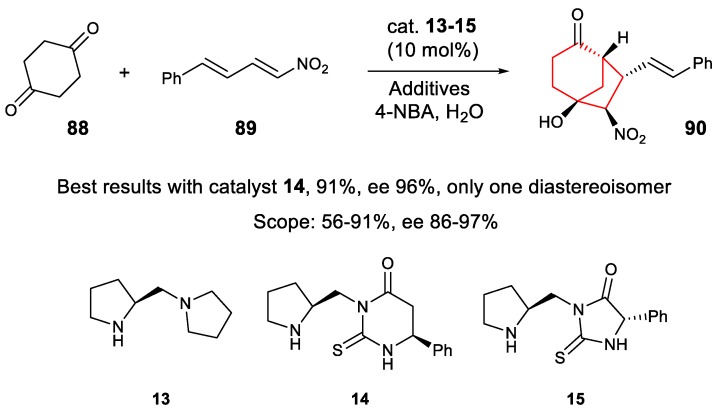
Domino Michael-Henry between 1,4-cyclohexadienone (**88**) and nitrodienes (**89**) allows the obtention of the bicyclic system: **90**.

Since the review of Rodriguez, there have been many contributions to this area starting with six membered rings. The groups of Kokotos and Ramachary have contributed specially to it. Kokotos starting with 1,4-cyclohexadione and unsaturated nitrodienes being able to obtain enantioenriched bicyclo[3.2.1]octan-2-ones by a domino Michael-Henry reaction using different organocatalysts as shown in Scheme 19 [39].

Kokotos group has probed several organocatalysts in the reaction of different ketones with nitrodienes. They observed that 1,4-cyclohexadienone with catalyst **16** with phenylnitrodiene gave the corresponding bicyclic compound in 48% yield, excellent diastereoselectivity (dr > 99:1) and a 97% ee [40].

**Scheme 20:**
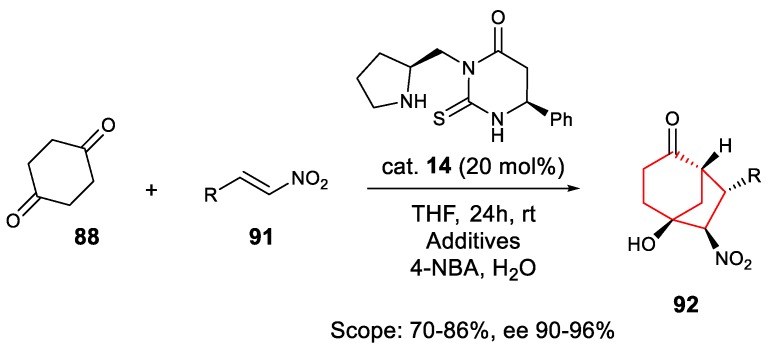
Domino Michael-Henry between 1,4-cyclohexadienone (**88**) and nitroolefins (**91**) yielding the product **92** with the bicyclo[3.2.1]octane core.

This group extended the scope of this methodology using nitroolefins, in this case, an extra quantity of the catalyst was necessary to obtain good results (Scheme 20) [39].

**Scheme 21:**
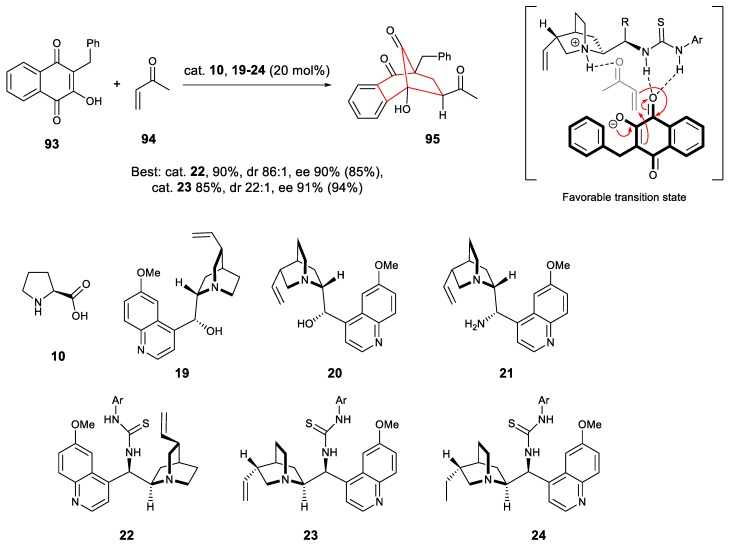
Domino Michael-aldol reaction between 2-benzyl-3-hidroxynaphtalene-1,4-dione **93** and methyl vinyl ketone **94** with different organocatalysts (**10** and **19**–**24**) producing the bicyclic system **95** with enantioselectivity via the transition state shown.

Recently, Ramachary et al. have achieved a versatile method to obtain methanobenzo[7]annulenes by organocatalytic asymmetric formal [3 + 2] cycloaddition, as can be seen in Scheme 21 [41].

**Scheme 22:**
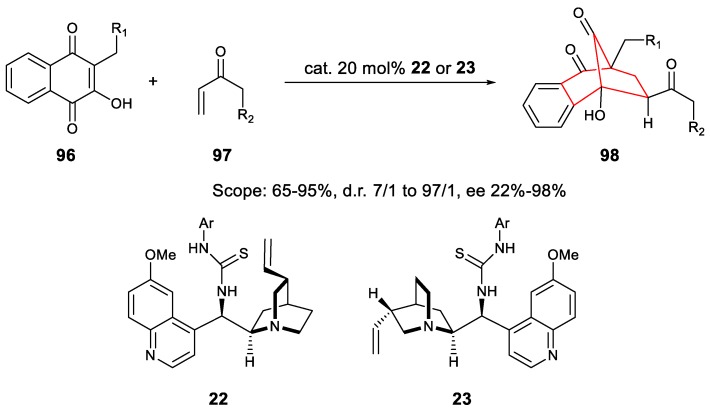
Domino Michael-aldol reaction between 2-akyl-3-hidroxynaphtalene-1,4-dione **96** and alkyl vinyl ketone **97** with different chiral organocatalysts allows the synthesis of the bicyclic product **98**.

This Michael-aldol reaction between methyl vinyl ketone and a hydroxydione was firstly assayed with a non-chiral basic catalyst achieving good diastereoselectivity and yield. The reaction catalyzed with chiral organocatalyst, proline (**10**), quinidine, or quinine based catalyst (**19**–**24**), determined that the best results were obtained when catalysts **22** and **23** were used. When the authors extended the methodology using 2-alkyl-3-hydroxynaphtalene-1,4-diones and methyl vinyl or ethyl vinyl ketone obtained in all case from good to excellent diastereoselectivities and enantiomeric excess using catalyst **22** or **23** (Scheme 22).

**Scheme 23:**
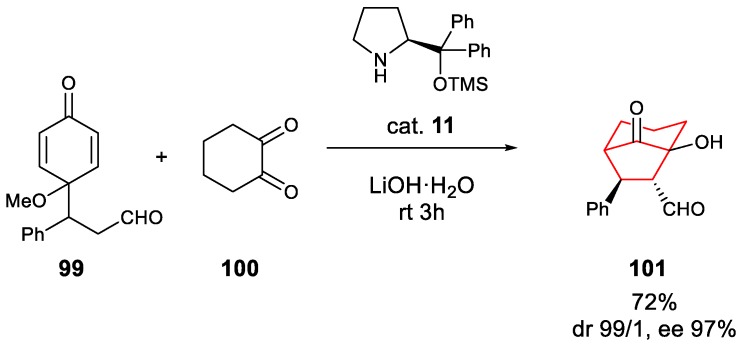
Enantioselective cyclization using a dienone (**99**) and a 1,2-diketone (**100**) produces **101**.

An interesting approach to bicyclo[3.2.1] systems has been carried out by Zhao who refers a methodology of an organocatalyzed C-C bond-scission reaction followed by the enantioselective β-alkylation of a 1,2-diketone as shown in Scheme 23 [42].

The authors demonstrated that firstly, the starting material (**99**) is transformed into the corresponding fenol and cinnamaldehyde that afterwards produces the corresponding enantioselective reaction by an enamine intermediate followed by a Michael-aldol cascade reaction to obtain the [3.2.1] bicyclic system **101**.

## 4. Conclusions

In summary, organic chemists have developed novel procedures for the synthesis of new chiral bicyclo[3.2.1]octanes. In this work, the synthesis of these compounds with chiral organocatalysts has been reviewed, recent synthesis with different functionalities have been reported and new methodologies have been presented for the synthesis of natural products in a very simple manner, as well as compounds with biological activities of special relevancy. The procedure reported by the group of Jørgensen, consisting on organocatalytic and stereoselective [6 + 4] cycloadditions, has opened the way for new skeletons of relevance in organic synthesis.

The use of bifunctional organocatalysts will be a new research area that in the next years will open new synthesis of a variety of compounds of this kind.

Non-chiral and chiral, alkaline and acid organocatalysts, have been summarized in this review. The acid organocatalyst consist in protonation-promoted rearrangement and are more limited than the use of basic organocatalyst that allows a more versatile reactivity: domino and aldol-like reactions.
